# Simultaneous heart-kidney transplantation outcomes in Asian populations in the United States: A united network for organ sharing database study

**DOI:** 10.1016/j.jhlto.2025.100364

**Published:** 2025-08-05

**Authors:** Shin Yajima, Hao He, Stefan Elde, Yuanjia Zhu, Y. Joseph Woo, Yasuhiro Shudo

**Affiliations:** a Department of Cardiothoracic Surgery, Stanford University, Stanford, CA; b Stanford Cardiovascular Institute, Stanford University, Stanford, CA

**Keywords:** Heart, Asia, Heart transplantation, Kidney transplantation, Renal Insufficiency

## Abstract

**Purpose:**

Simultaneous heart-kidney transplantation (HKTx) remains underutilized in regions outside the United States and Europe. Assessing the clinical outcomes of HKTx in Asian recipients is crucial for promoting its adoption in Asia. This retrospective study aimed to compare the survival outcomes of HKTx between Asian and non-Hispanic White (NHW) recipients with similar baseline characteristics.

**Methods:**

This study included 1494 recipients aged ≥18 years who underwent HKTx for the first time between 2000 and 2022. Among them, 1392 were NHW and 102 were Asian. Propensity score matching was used to balance the baseline characteristics between the Asian and NHW recipients. Kaplan–Meier survival analysis and log-rank tests were utilized to evaluate and compare overall survival.

**Results:**

In the prematched cohort, the in-hospital mortality rates were 10.0% and 6.7% for Asian and NHW recipients, respectively (p=0.216). Kaplan–Meier mortality estimates at 1, 5, and 10 years were 17.3%, 42.2%, and 67.7% for Asians, and 14.6%, 35.5%, and 65.9% for NHW recipients (p=0.692), respectively. After matching, the in-hospital mortality rates were 10.0% for Asians and 6.6% for NHW recipients (p=0.355). Long-term 1-, 5-, and 10-year mortality rates remained comparable: 13.9%, 36.1%, and 62.5% for Asians, versus 10.7%, 34.3%, and 59.2% for NHW recipients, respectively (p=0.665).

**Conclusion:**

Asian recipients demonstrated comparable long-term survival to NHW recipients after HKTx.

**Figure:**
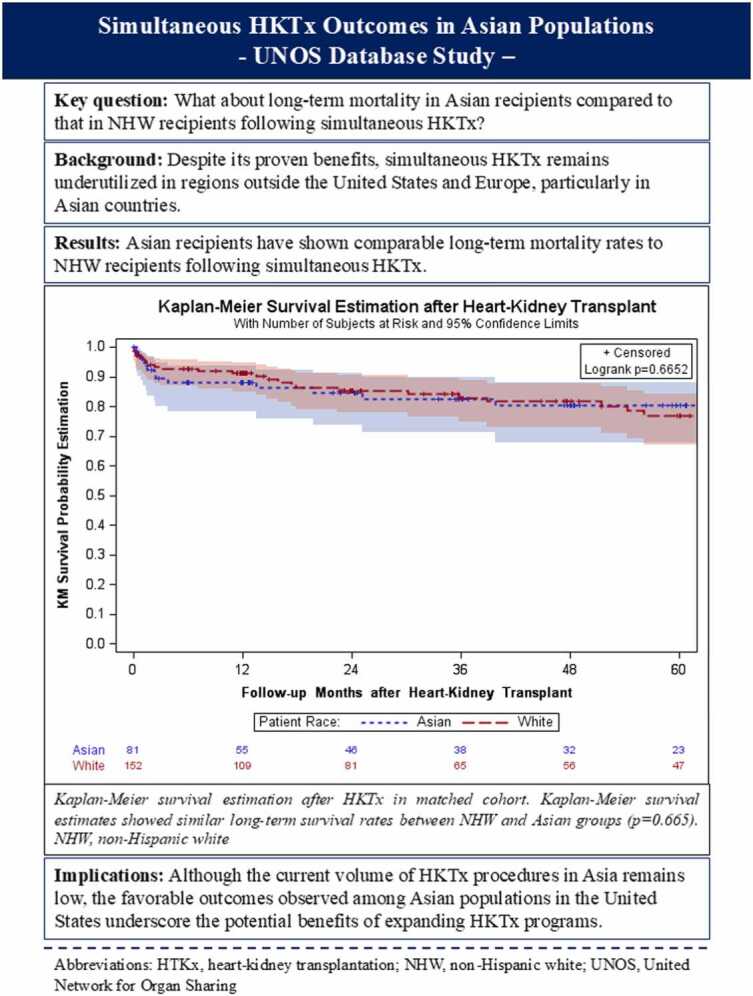

## Introduction

Over the past decade, simultaneous heart-kidney transplantation (HKTx) has emerged as the most common type of multiorgan transplantation, accounting for >85% of such procedures.^1^ Compared to heart transplantation alone, HKTx offers a significant survival advantage in patients with severe kidney dysfunction,^2^, ^3^, ^4^, ^5^ establishing it as a definitive strategy for patients with concurrent end-stage heart and kidney disease.

Despite its proven benefits, HKTx remains underutilized in regions outside the United States and Europe due to several challenges, including limited healthcare infrastructure, low organ donation rates, and cultural factors. A 2024 systematic review of simultaneous HKTx highlighted that most published studies (11/15) originated from the United States or Europe.^6^ Furthermore, 85.3% of multiorgan transplants worldwide were performed in the United States (US), compared to 12.6% in Europe and only 2.1% in other regions, including Asia.^1^ This disparity is particularly striking given that the Asia–Pacific region accounts for nearly 60% of the global population.^7^, ^8^ Therefore, understanding the clinical outcomes of HKTx in Asian recipients is critical to expanding its use in this region. To address this gap, this study aimed to investigate the outcomes of simultaneous HKTx in Asian recipients compared to non-Hispanic White (NHW) recipients with similar patient characteristics.

## Materials and methods

### Patient selection

This retrospective study analyzed deidentified data from the United Network for Organ Sharing (UNOS) database, obtained in its role as the contractor for the Organ Procurement and Transplant Network. The study cohort comprised patients aged >18 years who underwent first-time HKTx between January 2000 and 2022. Patients whose ethnicity was not identified as NHW or Asian were excluded. Race and ethnicity data were obtained from the UNOS database, where recipient ethnicity is registry-coded into seven predefined categories, including “Unknown.” For this study, we defined the "Asian" cohort by combining three categories: “Asian, Non-Hispanic,” “Alaska Native/American Indian, Non-Hispanic,” and “Native Hawaiian/Other Pacific Islander.” These classifications are derived from transplant center records at the time of listing and/or transplant and may include self-reported or clinician-assigned data. This study was approved as exempt by the institutional review board (IRB exemption number: 80203), as it involved deidentified data from the UNOS database. Additionally, the requirement for informed consent was waived.

For the recipients, we assessed the key demographic and clinical characteristics related to HKTx, including age, sex, sex matching, transplant year, blood type matching, body mass index (BMI), BMI ratio (recipient/donor), length of hospital stay, allograft ischemic time, distance from the donor hospital to transplant center, waiting list time, medical history (diabetes mellitus, dialysis, and malignancies), renal function, predicted heart mass, and the need for preoperative mechanical support (such as an intraaortic balloon pump [IABP] or extracorporeal membrane oxygenation [ECMO]).

For the donors, we primarily evaluated the factors that were available in the database, including age, sex, BMI, race, left ventricular ejection fraction, renal function, and comorbidities (cancer, diabetes, hypertension, and myocardial infarction).

This study compared the demographics and HKTx outcomes between the two recipient racial groups (NHW and Asian). The primary outcome was overall mortality.

### Statistical analysis

Continuous variables are presented as mean ± standard deviation or median with interquartile range and were compared using the Student’s *t*-test or Wilcoxon rank sum test, as appropriate. Categorical variables were compared using the Chi-squared test.

To better understand the survival outcomes, propensity score matching analysis was conducted to compare the NHW and Asian recipients. Propensity scores were calculated using logistic regression based on the race, incorporating the following covariates: recipient age, recipient gender, recipient/donor gender match, recipient/donor blood type match, recipient BMI, recipient/donor BMI ratio, donor BMI, estimated glomerular filtration rate (eGFR), recipient preoperative serum creatinine, recipient predicted heart mass (PHM), donor-recipient PHM mismatch, recipient diabetes, prior malignancies, hospital length of stay, ischemic time, distance from the donor hospital to transplant center, waiting list time, use of ECMO at the time of transplant, and mechanical support (including ventricular assist device [VAD]) at the time of transplant.

To address the covariate imbalance between the two groups, reduce bias from fixed matching ratios, and maximize data usage, **variable ratio matching was employed**, with matching ratios ranging from 2:1 to 1:1. This approach was chosen based on prior methodological work by Ming and Rosenbaum, who demonstrated that variable ratio matching can achieve substantially greater bias reduction compared to fixed-ratio matching, potentially eliminating up to 90% of covariate bias versus approximately 50% in 1:1 matching, even when using the same number of total controls.^9^, ^10^ Greedy matching without replacement was implemented using a caliper width of 0.005 to minimize propensity score distance and ensure similarity between the matched groups. After matching, standardized mean differences (SMDs) were calculated for all covariates, with an absolute SMD ≤ 0.25 considered indicative of acceptable balance.^11^, ^12^The primary outcome of overall survival was analyzed using Kaplan–Meier survival estimation, and survival curves were compared using the log-rank test. A two-sided alpha level of 0.05 was considered statistically significant for all analyses. Statistical analyses were performed using SAS version 9.4 (SAS Institute Inc., Cary, NC, USA).

## Results

### Prematched cohorts

**Figure 1:**
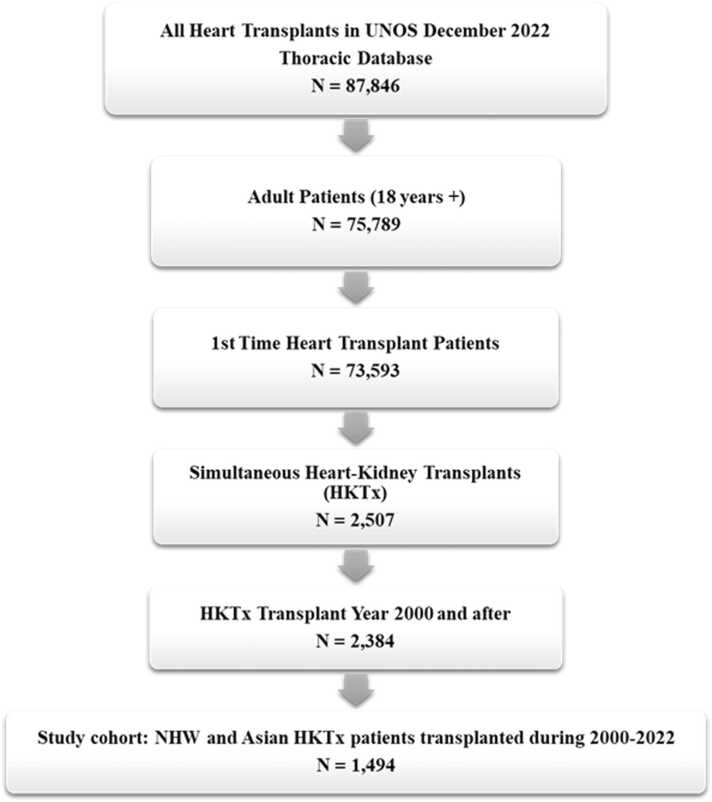
Patient selection. HKTx, heart-kidney transplantation; UNOS, United Network for Organ Sharing; NHW, non-Hispanic White.

A total of 1494 recipients aged >18 years who underwent first-time HKTx between 2000 and 2022 were identified, including 1392 NHW recipients and 102 Asian recipients (Figure 1). An analysis of the recipient demographics revealed no statistically significant difference between the two groups regarding age, gender, gender matching (recipient to donor), transplant year, blood type matching (recipient to donor), length of hospital stay, allograft ischemic time, and distance from the donor hospital to transplant center. However, Asian recipients had a lower BMI and shorter waiting list time than NHW recipients.

Regarding the recipient conditions prior to transplantation, the rates of previous cardiac and lung surgeries, as well as the use of mechanical support, such as IABPs, ventilators, and VAD, were not statistically significant different between the two groups. However, the indications for heart transplantation differed: Asian recipients had predominantly ischemic and nonischemic etiologies, with no cases of congenital or restrictive etiologies. In contrast, NHW recipients had a wider range of etiologies, including congenital and restrictive causes, in addition to ischemic and nonischemic causes.

Regarding the medical history, Asian recipients had a lower prevalence of malignancy, but a higher prevalence of diabetes and dialysis dependency compared with NHW recipients. Renal function was more impaired in Asian than in NHW recipients. Additionally, Asian recipients had a smaller PHM, leading to a higher rate of donor-recipient PHM mismatch, as well as greater reliance on ECMO before HKTx (Table 1).

**Table 1:** Prematched Baseline Characteristics of the Recipients

	Total Cohort, N=1494	NHW, n=1392	Asian, n=102	p-value^a^
Recipient demographics				
Age (y)	Mean±SD	57.0±9.9	55.2±10.7	0.079
Gender	Female (%)	239 (17.2%)	17 (16.7%)	0.897
	Male (%)	1153 (82.8%)	85 (83.3%)	
Gender match (recipient to donor)	Yes (%)	1074 (77.2%)	72 (70.6%)	0.129
Body surface area	Mean±SD	1.98±0.23	1.80±0.20	<0.001
Transplant year	2000–2004	93 (6.7%)	3 (2.9%)	0.165
	2005–2009	152 (10.9%)	8 (7.8%)	
	2010–2014	199 (14.3%)	10 (9.8%)	
	2015–2019	445 (32.0%)	41 (40.2%)	
	2020–2022	503 (36.1%)	40 (39.2%)	
Blood type match	Yes (%)	1185 (85.1%)	83 (81.4%)	0.307
(recipient to donor)				
BMI (kg/m^2^)	Mean±SD	27.1±4.9	24.7±4.4	<0.001
BMI ratio (recipient/donor)	Mean±SD	1.03±0.23	0.95±0.25	0.003
Length of hospital stay (days)	Median [IQR]	20 (14–32)	18 (13–33)	0.364
Allograft ischemic time (hrs)	Mean±SD	3.3±1.0	3.4±1.2	0.368
Distance, donor hospital to transplant center (miles)	Median [IQR]	105 [19–282.5]	104 [22–264]	0.867
Time on waitlist (days)	Median [IQR]	63 [19–200]	44.5 [10–124]	0.006
Preoperative recipient transplantation conditions				
Indication for heart transplantation	Congenital	27 (1.9%)	0 (0.0%)	0.004
	Hypertrophic	41 (3.0%)	4 (3.9%)	
	Ischemic	572 (41.1%)	48 (47.1%)	
	Nonischemic	532 (38.2%)	40 (39.2%)	
	Restrictive	67 (4.8%)	0 (0.0%)	
	Valvular	27 (1.9%)	1 (1.0%)	
	Other	126 (9.1%)	9 (8.9%)	
Previous cardiac surgery	Yes (%)	644 (49.3%)	42 (41.6%)	0.135
(nontransplant)				
Previous lung surgery	Yes (%)	9 (0.7%)	0 (0.0%)	0.989
Previous kidney surgery				
Any malignancies prior to transplantation	Yes (%)	144 (10.4%)	5 (4.9%)	<0.001
Diabetes prior to transplantation	Yes (%)	602 (43.6%)	70 (68.6%)	<0.001
History of dialysis prior to transplantation	Yes (%)	627 (45.9%)	59 (58.4%)	0.015
Recipient serum creatinine (mg/dL)	Median [IQR]	2.4 [1.8–3.5]	2.7 [1.8–5.2]	0.033
eGFR	Median [IQR]	26.9 [17.4–38.2]	21.8 [10.5–38.6]	0.039
PHM (g)	Median [IQR]	184.8 [162.5–206.2]	165.7 [149.7–182.1]	<0.001
Donor-recipient PHM mismatch (% difference)	Median [IQR]	0.4 [−9.4, 11.9]	6.3 [−3.8, 19.4]	0.001
Mechanical circulatory support at transplant				
ECMO	Yes (%)	39 (2.8%)	4 (3.9%)	<0.001
IABP	Yes (%)	219 (15.7%)	16 (15.7%)	0.991
Ventilator	Yes (%)	24 (1.7%)	1 (1.0%)	0.572
VAD	Yes (%)	433 (31.1%)	28 (27.5%)	0.441
Mechanical support (including VAD)	Yes (%)	1059 (77.4%)	69 (68.3%)	0.038

BMI, body mass index; ECMO, extracorporeal membrane oxygenation; eGFR, estimated glomerular filtration rate; IABP, intraaortic balloon pump; IQR, interquartile range; NHW, non-Hispanic White; PHM; predicted heart mass; SD, standard deviation; VAD: ventricular assist device

a p-values obtained using the Chi-squared test, Fisher's exact test, two-sample t-test, or Wilcoxon rank sum test

**Figure 2:**
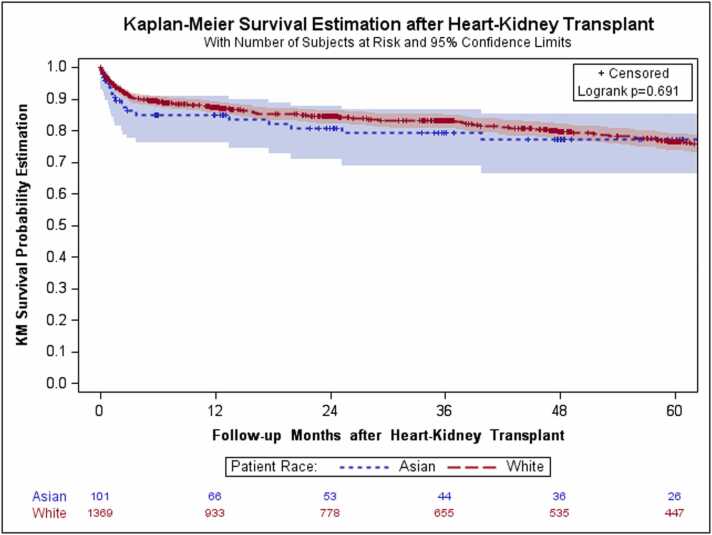
Kaplan–Meier survival analysis after heart-kidney transplantation in the prematched cohort. Kaplan–Meier survival estimates show no statistically significant difference in the long-term survival rates between the NHW and Asian groups (p=0.692). Log-rank test was used to compare survival between the groups. Censoring marks are indicated. NHW, non-Hispanic White.

Donor demographics, including age, gender, BMI, race, heart and kidney function, cause of death, and comorbidities, were not statistically significant different between the two groups (Table 2). Regarding the operative outcomes, the in-hospital mortality rates were 10.0% and 6.7% for Asian and NHW recipients, respectively (p=0.216). The incidence of postoperative complications, such as stroke, dialysis, pacemaker implantation, and primary graft failure, was not statistically significant different between the two groups. Retransplantation rates, including isolated heart, isolated kidney, and simultaneous HKTx, were also not statistically significant different. Kaplan–Meier survival estimates at 1, 5, and 10 years post-HKTx showed no statistically significant differences in long-term survival rates between the two groups: 17.3%, 42.2%, and 67.7% for Asian recipients and 14.6%, 35.5%, and 65.9% for NHW recipients, respectively (p=0.692, Table 3 and Figure 2).

**Table 2:** Prematched Baseline Characteristics of the Donors

	Total Cohort, N=1494	NHW, n=1392	Asian, n=102	p-value^a^
Donor demographics				
Age (y)	Mean±SD	32.1±11.1	33.2±11.6	0.331
Gender	Female (%)	355 (25.5%)	33 (32.4%)	0.128
	Male (%)	1037 (74.5%)	69 (67.7%)	
Body surface area	Mean±SD	1.97±0.22	1.91±0.24	0.004
Race	White (%)	1188 (85.3%)	84 (82.4%)	0.171
	Black (%)	167 (12.0%)	12 (11.8%)	
	Others (%)	37 (2.7%)	6 (5.9%)	
Left ventricular ejection fraction (%)	Mean±SD	61.9±7.1	62.2±6.4	0.714
Creatinine (mg/dL)	Mean±SD	1.04±0.66	0.98±0.42	0.411
Cause of Death				0.157
Anoxia		433 (31.1%)	27 (26.5%)	
Cerebrovascular accident		217 (15.6%)	24 (23.5%)	
Head trauma		708 (50.9%)	47 (46.1%)	
Other		34 (2.4%)	4 (3.9%)	
Donor comorbidities				
Cancer	Yes (%)	22 (1.6%)	3 (2.9%)	0.581
Diabetes	Yes (%)	31 (2.2%)	2 (2.0%)	0.563
Hypertension	Yes (%)	167 (12.0%)	15 (14.7%)	0.716
Myocardial infarction	Yes (%)	9 (0.7%)	0 (0.0%)	0.425

NHW, non-Hispanic White; SD, standard deviation.

a p-values from the Chi-squared test, Fisher's exact test, two-sample t-test, or Wilcoxon rank sum test.

**Table 3:** Prematched Posttransplant Outcomes of the Recipients

	Total Cohort, N=1494	NHW, n=1392	Asian, n=102	p-value^a^
Stroke	Yes (%)	45 (3.3%)	3 (3.0%)	0.582
Dialysis	Yes (%)	425 (31.1%)	27 (26.7%)	0.433
Pacemaker implantation	Yes (%)	28 (2.1%)	1 (1.0%)	0.501
Primary graft failure - Heart	Yes (%)	371 (27.1%)	26 (25.7%)	0.764
Received isolated heart transplantation after initial transplantation	Yes (%)	2 (0.1%)	0 (0.0%)	0.999
Received isolated kidney transplantation after initial transplantation	Yes (%)	11 (0.8%)	1 (1.0%)	0.573
Received simultaneous HKTx after initial transplantation	Yes (%)	4 (0.3%)	0 (0.0%)	0.999
In-hospital mortality	Yes (%)/N	92 (6.7%)/1366	10 (10.0%)/100	0.216
1-year mortality	Yes (%)/N	162 (14.6%)/1108	14 (17.3%)/81	0.515
5-year mortality	Yes (%)/N	249 (35.3%)/705	19 (42.2%)/45	0.349
10-year mortality	Yes (%)/N	335 (65.9%)/508	21 (67.7%)/31	0.835
Survival/Follow-up Time by 12/31/2022 (days)	Median [IQR]	1084 [320–2217]	750 [114–1834]	0.123

HKTx, heart-kidney transplantation; IQR, interquartile range; NHW, non-Hispanic White

a p-values from the Chi-squared test, Fisher's exact test, two-sample t-test, or Wilcoxon rank sum test

To further evaluate the impact of pre-transplant renal function on post-HKTx survival, we conducted a stratified analysis based on eGFR. Patients were categorized into three strata: eGFR < 30, 30–45, and > 45 mL/min/1.73 m² (Supplemental Table 1). Within each eGFR group, we compared mortality outcomes (in-hospital, 1-year, 5-year, and 10-year) between Asian and NHW recipients. Across all eGFR strata and time points, no statistically significant differences in mortality were observed between the two groups (all p > 0.1 by Fisher’s exact test). The results are summarized in Supplemental Table 2 and Supplemental Figure 1.

### Matched cohorts

**Figure 3:**
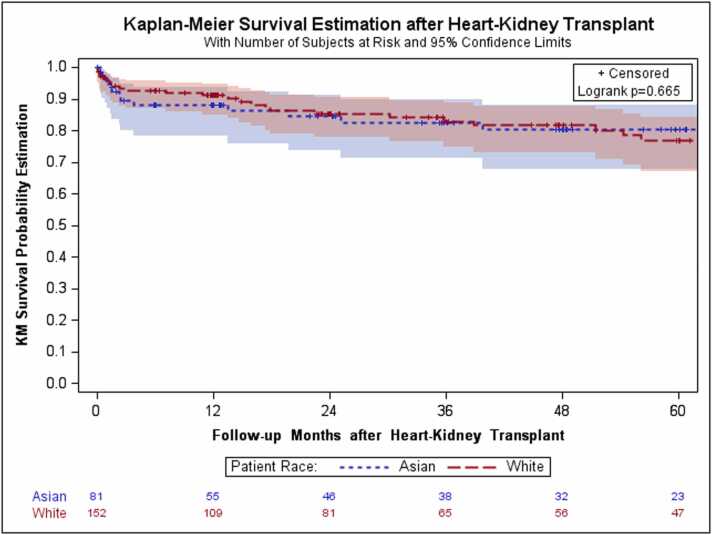
Kaplan–Meier survival estimation after heart-kidney transplantation in the matched cohort. Kaplan–Meier survival estimates show no statistically significant difference in the long-term survival rates between the NHW and Asian groups (p=0.665). Log-rank test was used to compare survival between the groups. Censoring marks are indicated. NHW, non-Hispanic White.

The SMDs of all covariates were well-balanced with all absolute SMDs ≤0.25 (Supplemental Table 3 and Supplemental Figure 2). After propensity score matching, a total of 233 recipients were identified, including 152 NHW recipients and 81 Asian recipients. Baseline characteristics and perioperative variables of the recipients and donors are summarized in Supplemental Tables 4 and 5, respectively. Regarding the operative outcomes, the in-hospital mortality rate was 10.0% for Asian recipients and 6.6% for NHW recipients (p=0.355). Postoperative complications and retransplantation rates were not statistically significant different between the groups. Kaplan–Meier survival estimates at 1, 5, and 10 years post-HKTx showed no statistically significant difference in long-term mortality rates between the two groups: 13.9%, 36.1%, and 62.5% for Asian recipients and 10.7%, 34.3%, and 59.2% for NHW recipients, respectively (p=0.665, Table 4 and Figure 3).

**Table 4:** Matched Posttransplant Outcomes of the Recipients

	Total Matched Cohort, N=233	Matched NHW, n=152	Matched Asian, n=81	p-value^a^
Stroke	Yes (%)	5 (3.3%)	1 (1.2%)	0.668
Dialysis	Yes (%)	44 (29.0%)	22 (27.2%)	0.773
Pacemaker implantation	Yes (%)	2 (1.3%)	0 (0.0%)	0.545
Primary graft failure - heart	Yes (%)	34 (22.4%)	20 (24.7%)	0.689
Received isolated heart transplant after initial transplant	Yes (%)	0 (0.0%)	0 (0.0%)	NA
Received isolated kidney transplant after initial transplant	Yes (%)	1 (0.7%)	1 (1.2%)	0.999
Received simultaneous HKTx	Yes (%)	0 (0.0%)	0 (0.0%)	NA
In-hospital mortality	Yes (%)/N	10 (6.6%)/152	8 (10.0%)/80	0.355
1-year mortality	Yes (%)/N	13 (10.7%)/122	9 (13.9%)/65	0.519
5-year mortality	Yes (%)/N	25 (34.3%)/73	13 (36.1%)/36	0.848
10-year mortality	Yes (%)/N	29 (59.2%)/49	15 (62.5%)/24	0.786
Survival/follow-up time by 12/31/2022 (days)	Median [IQR]	843.5 [362.5–2194.5]	1077 [181–1855]	0.733

HKTx, heart-kidney transplantation; IQR, interquartile range; NHW, non-Hispanic White

a p-values from the Chi-squared test, Fisher's exact test, two-sample t-test, or Wilcoxon rank sum test

## DISCUSSION

This study clearly demonstrates that simultaneous HKTx achieves comparable long-term survival outcomes in Asian recipients relative to NHW recipients in both prematched and propensity score–matched cohorts. Importantly, Asian ethnicity was not identified as an independent risk factor for inferior long-term survival. These findings resonate with previous studies in the field of solid organ transplantation, including heart transplantation and combined heart-lung transplantation, that have shown non-inferior long-term outcomes among Asian recipients when baseline characteristics are appropriately adjusted.^13^, ^14^

Kaplan–Meier survival analyses further support this observation. In both the prematched and matched cohorts, the survival curves of Asian and NHW recipients remained closely aligned throughout the follow-up period, with no statistically significant divergence at 1, 5, or 10 years. Censoring marks were evenly distributed between the groups, and the number-at-risk tables confirmed adequate follow-up across time intervals. The lack of visual and statistical separation reinforces the conclusion that Asian ethnicity is not associated with inferior post-transplant survival following HKTx in the US.

Notably, Asian recipients in this study exhibited poorer preoperative kidney function in the prematched cohort, with a higher prevalence of diabetes mellitus, dialysis dependence, and lower eGFR, all factors previously associated with adverse outcomes following HKTx.^2^, ^15^, ^16^, ^17^, ^18^ Despite these risk factors, survival outcomes remained comparable to NHW recipients. Although the specific mechanisms underlying this outcome cannot be definitively identified using the UNOS dataset, our results suggest that Asian patients are not disadvantaged in terms of post-transplant survival when provided equitable access to care. No large-scale multicenter study to date has definitively assessed race or ethnicity as independent determinants of HKTx outcomes.^19^

In addition, we conducted a stratified analysis by baseline eGFR (<30, 30–45, and >45 mL/min/1.73m²) to further explore whether impaired renal function modified the effect of race on survival. Across all renal function categories, no statistically significant differences in mortality were observed between Asian and NHW recipients at the in-hospital, 1-year, 5-year, or 10-year timepoints. Although the sample size of Asian recipients was limited within each stratum, particularly in the higher eGFR group, this analysis further supports the generalizability of our findings across clinically relevant subgroups.

Our findings also build upon a 2024 systematic review,^6^ which summarized the outcomes of multiorgan transplantation across various racial and ethnic groups. While the review highlighted a general lack of race-specific evidence for simultaneous HKTx, especially among Asian populations, our study directly addressed this gap by conducting the first matched cohort analysis specifically comparing Asian and NHW recipients. Unlike the review, which was descriptive and limited by heterogeneity across centers and study designs, our analysis used standardized national registry data and rigorous propensity score matching. This allowed for a more robust, population-based comparison of long-term survival outcomes in a previously underrepresented group.

While our findings are based on a US population, they raise important questions regarding the generalizability of these outcomes to Asian countries. Asians living in the US often receive care in high-resource environments with standardized perioperative protocols and access to specialized transplant centers. In contrast, the transplant infrastructure, access to organs, and post-transplant care vary widely across Asian countries. For instance, the overall organ transplantation rate in Southeast Asia remains markedly lower, approximately 3.8 transplants per million population, compared to 31.6 in the US and 27.9 in Europe.^20^ This disparity is even more pronounced for multiorgan transplantation such as HKTx, which is performed only in a limited number of high-volume centers in Asia. Japan, for example, has yet to report any cases of HKTx, in part due to cultural barriers to organ donation and historical limitations in legal frameworks surrounding brain death.^21^ In China and India, two countries that account for a substantial proportion of the global burden of chronic kidney disease,^22^ HKTx remains uncommon and is largely restricted to tertiary care centers. While these differences present clear limitations to the direct generalization of our findings, the observed success rates in Asian recipients in the US suggests that favorable outcomes may be achievable in Asian countries when similar levels of care and access are established.

In addition, the UNOS database uses broad, registry-defined race and ethnicity categories that may conflate diverse subpopulations. Our definition of “Asian” included recipients classified as Asian, Native Hawaiian/Other Pacific Islander, and Alaska Native/American Indian. These groups encompass heterogeneous genetic, cultural, and geographic backgrounds, which may limit the interpretability of our findings across specific subgroups. Furthermore, pharmacogenomic variability, such as polymorphisms in *CYP3A5* affecting tacrolimus metabolism, can significantly influence immunosuppressive drug levels and efficacy in different Asian populations.^23^ Cultural attitudes, dietary practices, and healthcare access may also differ between Asians residing in the US and those living in Asia, further complicating direct extrapolation.

Nonetheless, our study sets a meaningful precedent for the expansion of HKTx in Asia. As the prevalence of advanced heart failure and chronic kidney disease continues to rise across the region, estimated to affect more than 434 million individuals in eastern, southern, and southeastern Asia, particularly in China and India,^22^ there is a growing imperative to develop dual-organ transplant programs. The comparable survival outcomes observed in this study underscore the potential benefits of implementing HKTx more broadly in Asia, particularly in high-risk patients with combined end-stage heart and kidney disease. Achieving this goal will require overcoming systemic barriers, including legal and cultural constraints, donor shortages, and infrastructure limitations.

This study has several other limitations. First, the analysis relied heavily on propensity score matching, which was constrained to variables with sufficient completeness in the UNOS dataset. As a retrospective registry study, we lacked information on critical variables such as dialysis duration and modality, immunosuppressive protocols, and rejection episodes. These unmeasured factors may have influenced both short- and long-term outcomes. For example, the clinical trajectory and prognosis of patients undergoing chronic outpatient dialysis likely differ substantially from those requiring brief perioperative dialysis for acute optimization, yet this distinction could not be assessed in our dataset. Similarly, the inability to evaluate variations in immunosuppressive induction or maintenance therapy prevented us from fully interpreting the mechanisms behind the observed survival rates. These limitations underscore the need for additional granular, prospective data to delineate these effects more precisely. Second, the sample size of Asian recipients, particularly in the matched cohort, was relatively small, which may have limited the statistical power to detect subtle differences and increased the risk of Type II error. Although we considered conducting a post hoc power analysis, we ultimately chose not to include it given the consensus in the statistical literature that such analyses are often misleading in retrospective studies and add limited interpretive value, especially when the effect sizes and p-values are already reported.^24^, ^25^, ^26^, ^27^ Finally, future studies evaluating same-ethnicity donor-recipient matching, such as Asian-to-Asian versus NHW-to-NHW transplants, may further clarify the impact of race and ethnicity on long-term transplant outcomes.

Despite these limitations, this study provides the first robust evidence that Asian recipients of simultaneous HKTx can achieve survival outcomes equivalent to NHW recipients in the US. These results provide a valuable benchmark for Asian countries aiming to expand dual-organ transplant programs and highlight the need for further region-specific investigations. With appropriate allocation systems, clinical infrastructure, and societal engagement, similar outcomes may be attainable in Asia, supporting the global equity and scalability of multiorgan transplantation.

## Conclusion

Asian recipients demonstrated long-term mortality rates comparable to those of NHW recipients following simultaneous HKTx, supporting the potential feasibility and clinical value of broader adoption of this procedure in Asian countries. Although the current volume of HKTx procedures in Asia remains low, the favorable outcomes observed in the Asian population in the US underscore the potential benefits of expanding HKTx programs. Overcoming cultural, legal, and logistical challenges will be essential for improving the quality of care and survival outcomes of high-risk patients in Asian countries.

## Research ethics

This study was exempted from ethical review by the institutional review board of our institution because the UNOS database contains no patient identifiers. Additionally, the requirement for informed consent was waived.

## Funding

This study was supported by the Department of Cardiothoracic Surgery’s DEI program at Stanford university.

## CRediT authorship contribution statement

SY: Conceptualization, Visualization, Writing – original draft, Project Administration; HH: Formal analysis, Investigation, Data Curation, Writing – original draft; SE: Writing – review and editing; YZ: Writing – review and editing; YJW: Writing – review and editing, Supervision; YS: Validation, Writing – review and editing, Supervision, Project administration.

## Data Availability

All relevant data supporting the findings of this study are within the article and its Supplemental Information files. Additional analyses or supporting datasets are available from the authors upon request.

## Declaration of Competing Interest

The authors declare that they have no known competing financial interests or personal relationships that could have appeared to influence the work reported in this paper.

## Glossary

BMI: body mass index
ECMO: extracorporeal membrane oxygenation
eGFR: estimated glomerular filtration rate
IABP: intraaortic balloon pump
KAH: kidney after heart transplantation
NHW: non-Hispanic White
PHM: predicted heart mass
SHKT: simultaneous heart-kidney transplantation
SMD: standardized mean difference
UNOS: United Network for Organ Sharing
US: United States
VAD: ventricular assist device

## Footnotes

Appendix A Supplemental data associated with this article can be found in the online version at doi:10.1016/j.jhlto.2025.100364.
