# Supplementary material for: Simultaneous heart-kidney transplantation outcomes in Asian populations in the United States: A united network for organ sharing database study
Source: JHLT Open. 2025 Aug 5;10:100364. doi: 10.1016/j.jhlto.2025.100364 (PMC12396451; doi:10.1016/j.jhlto.2025.100364)
Supplement: Supplementary file 1 — Supplemental material [file mmc1.docx]

**Simultaneous heart-kidney transplantation outcomes in Asian populations: A United Network for Organ Sharing database study**

Shin Yajima, MD, PhD^a,b^; Hao He, PhD^a^; Stefan Elde, MD^a,b^; Yuanjia Zhu, MD, PhD^a,b^; Y. Joseph Woo, MD^a,b^; Yasuhiro Shudo, MD, PhD^a,b^

^a^Department of Cardiothoracic Surgery, Stanford University, Stanford, CA, USA

^b^Stanford Cardiovascular Institute, Stanford University, Stanford, CA, USA

**ORCIDs**

Shin Yajima: https://orcid.org/0000-0001-5058-0267

Hao He: https://orcid.org/0000-0001-7029-6992

Stefan Elde: https://orcid.org/0000-0001-7103-6148

Yuanjia Zhu: https://orcid.org/0000-0001-5642-9883

Y. Joseph Woo: N/A

Yasuhiro Shudo: https://orcid.org/0000-0002-4545-1273

**Correspondence**: Yasuhiro Shudo, MD, PhD, Clinical Assistant Professor

Department of Cardiothoracic Surgery, Stanford University School of Medicine, 870 Quarry Road, Palo Alto, CA 94304

Email: yshudo@stanford.edu

**Supplementary Table 1. eGFR group distribution by race**

| eGFR Group | Total N (column%) | Race | | |
| --- | --- | --- | --- | --- |
|  |  | **NHW, n (row %)** | | **Asian, n (row %)** |
| Total N (row %) | 1464* | 1364 (93.7%) | | 100 (6.8%) |
| eGFR < 30 | 842 (57.5%) | 779 (92.5%) | | 63 (7.5%) |
| eGFR 30-45 | 368 (25.1%) | 347 (94.3%) | | 21 (5.7%) |
| eGFR > 45 | 254 (17.4%) | 238 (93.7%) | | 16 (6.3%) |
|  |  |  | |  |
| * Frequency Missing = 30 |  |  |  | |

eGFR, estimated glomerular filtration rate; NHW, non-Hispanic White

**Supplementary Table 2. In-hospital, 1-year, 5-year, and 10-year mortality across all eGFR groups, further stratified by race (NHW vs. Asian)**

| **Mortality Outcome** | **eGFR Group** | **N** | **NHW** | | **Asian** | | **p-value from Fisher's Exact Test** |
| --- | --- | --- | --- | --- | --- | --- | --- |
|  |  |  | **Total NHW, n** | **Mortality, n (%)** | **Total Asian, n** | **Mortality n (%)** |  |
| **In-hospital mortality** | eGFR < 30 | 839 | 777 | 52 (6.7%) | 62 | 7 (11.3%) | 0.191 |
|  | eGFR 30-45 | 367 | 346 | 19 (5.5%) | 21 | 3 (14.3%) | 0.123 |
|  | eGFR > 45 | 253 | 237 | 21 (8.9%) | 16 | 0 (0.0%) | 0.375 |
| **1-year mortality** | eGFR < 30 | 699 | 648 | 94 (14.5%) | 51 | 10 (19.6%) | 0.309 |
|  | eGFR 30-45 | 283 | 266 | 34 (12.8%) | 17 | 4 (23.5%) | 0.259 |
|  | eGFR > 45 | 201 | 188 | 33 (17.6%) | 13 | 0 (0.0%) | 0.132 |
| **5-year mortality** | eGFR < 30 | 461 | 427 | 142 (33.3%) | 34 | 14 (41.2%) | 0.352 |
|  | eGFR 30-45 | 160 | 153 | 54 (35.3%) | 7 | 4 (57.1%) | 0.256 |
|  | eGFR > 45 | 125 | 121 | 52 (43.0%) | 4 | 1 (25.0%) | 0.637 |
| **10-year mortality** | eGFR < 30 | 331 | 309 | 197 (63.8%) | 22 | 16 (72.7%) | 0.493 |
|  | eGFR 30-45 | 118 | 112 | 72 (64.3%) | 6 | 4 (66.7%) | 0.999 |
|  | eGFR > 45 | 86 | 83 | 64 (77.1%) | 3 | 1 (33.3%) | 0.146 |

**eGFR, estimated glomerular filtration rate; NHW, non-Hispanic White**

**Supplementary Table 3. SMDs of NHW vs. Asian recipients**

| **Matching Factors** | **SMDs for all recipients before matching** | **SMDs for the matched groups** |
| --- | --- | --- |
|  |  |  |
| Recipient age | -0.187 | -0.002 |
| Recipient/donor gender match | 0.072 | -0.009 |
| Recipient/donor blood type match | 0.047 | 0.026 |
| Recipient BMI | -0.570 | 0.062 |
| Recipient/donor BMI ratio | -0.276 | -0.017 |
| eGFR score before transplant | 0.011 | 0.063 |
| Recipient preop serum creatinine | 0.291 | 0.019 |
| Recipient PHM | -0.687 | 0.003 |
| Donor PHM | -0.301 | -0.049 |
| Donor-recipient PHM mismatch | 0.323 | -0.048 |
| Recipient diabetes | -0.243 | -0.015 |
| Recipient any malignancies prior transplantation | 0.054 | 0.011 |
| Recipient length of hospital stay | -0.001 | 0.001 |
| Ischemic time | 0.084 | 0.013 |
| Distance, donor hospital to transplant center | 0.049 | 0.071 |
| Days on waiting list | -0.270 | -0.006 |
| ECMO at transplant | -0.016 | -0.011 |
| Mechanical support (including VAD) at transplant | 0.087 | 0.033 |

BMI, body mass index; ECMO, extracorporeal membrane oxygenation; eGFR, estimated glomerular filtration rate; NHW, non-Hispanic White, PHM, predicted heart mass; SHKT, simultaneous heart-kidney transplantation; SMD, standardized mean difference; VAD, ventricular assist device

**Supplementary Table 4. Matched baseline characteristics of the recipients**

|  | **Total Matched Cohort, N=233** | **Matched NHW n=152** | **Matched Asian n=81** | **p-value^a^** |
| --- | --- | --- | --- | --- |
|  |  |  |  |  |
| **Recipient demographics** | | | | |
| Age (y) | Mean ± SD | 56.8±10.0 | 56.5±9.4 | 0.874 |
| Gender | Female (%) | 24 (15.8%) | 12 (14.8%) | 0.845 |
|  | Male (%) | 128 (84.2%) | 69 (85.2%) |  |
| Gender match (recipient to donor) | Yes (%) | 115 (75.7%) | 62 (76.5%) | 0.880 |
| Body surface area | Mean ± SD | 1.84±0.18 | 1.83±0.18 | 0.888 |
| Transplant year | 2000–2004 | 8 (5.3%) | 3 (3.7%) | 0.947 |
|  | 2005–2009 | 17 (11.2%) | 7 (8.6%) |  |
|  | 2010–2014 | 15 (9.9%) | 8 (9.9%) |  |
|  | 2015–2019 | 54 (35.5%) | 31 (38.3%) |  |
|  | 2020–2022 | 58 (38.2%) | 32 (39.5%) |  |
| Blood type match (recipient to donor) | Yes (%) | 124 (81.6%) | 64 (79.0%) | 0.637 |
| Recipient BMI (kg/m^2^) | Mean ± SD | 24.8±4.0 | 25.1±4.2 | 0.649 |
| BMI ratio (recipient/donor) | Mean ± SD | 0.97±0.21 | 0.97±0.26 | 0.906 |
| Length of hospital stay (days) | Median [IQR] | 20 [14–29] | 18 [14–33] | 0.758 |
| Allograft ischemic time (hours) | Mean ± SD | 3.4±1.0 | 3.4±1.3 | 0.929 |
| Distance, donor hospital to transplant center (miles) | Median [IQR] | 95 [19–226] | 83 [21–264] | 0.882 |
| Time on waitlist (days) | Median [IQR] | 39 [18–104.5] | 45 [7–124] | 0.522 |
| **Preoperative recipient conditions** | | | | |
| Indication for heart transplant | Hypertrophic | 4 (2.6%) | 3 (3.7%) | 0.853 |
|  | Ischemic | 77 (50.7%) | 41 (50.6%) |  |
|  | Nonischemic | 64 (42.1%) | 30 (37.0%) |  |
|  | Others | 7 (4.6%) | 7 (8.7%) |  |
| Previous cardiac surgery (nontransplant) | Yes (%) | 66 (45.8%) | 33 (41.3%) | 0.508 |
| Previous lung surgery | Yes (%) | 1 (0.7%) | 0 (0.0%) | 0.989 |
| Previous kidney surgery |  |  |  |  |
| Any malignancies prior to transplant | Yes (%) | 11 (7.2%) | 5 (6.2%) | 0.759 |
| Diabetes prior to transplant | Yes (%) | 101 (66.5%) | 55 (67.9%) | 0.822 |
| History of dialysis prior to transplant | Yes (%) | 78 (51.7%) | 45 (55.6%) | 0.571 |
| Recipient serum creatinine (mg/dL) | Median [IQR] | 2.6 [1.8–4.4] | 2.7 [1.8–4.6] | 0.944 |
| eGFR | Median [IQR] | 24.3 [13.5–38.1] | 24.5 [13.4–38.7] | 0.939 |
| PHM (grams) | Median [IQR] | 172.0 [157.3–183.8] | 171.2 [155.7–183.6] | 0.912 |
| Donor-recipient PHM mismatch (% difference) | Median [IQR] | 7.2 [-2.3, 18.9] | 7.6 [-4.5, 19.5] | 0.626 |
| Mechanical circulatory support at transplant | | | | |
| ECMO | Yes (%) | 2 (1.3%) | 2 (2.5%) | 0.612 |
| IABP | Yes (%) | 33 (21.7%) | 15 (18.5%) | 0.566 |
| Ventilator | Yes (%) | 3 (2.0%) | 1 (1.2%) | 0.679 |
| VAD | Yes (%) | 44 (29.0%) | 23 (28.4%) | 0.929 |
| Mechanical support (including VAD) | Yes (%) | 112 (73.7%) | 57 (70.4%) | 0.589 |

BMI, body mass index; ECMO, extracorporeal membrane oxygenation; eGFR, estimated glomerular filtration rate; IABP, intraaortic balloon pump; IQR, interquartile range; NHW, non-Hispanic White; PHM, predicted heart mass; SD, standard deviation; VAD: ventricular assist device

^a^ p-values from Chi-squared test, Fisher's exact test, two-sample t-test, or Wilcoxon rank sum test

**Supplementary Table 5. Matched baseline characteristics of donors**

|  | **Total Matched Cohort, N=233** | | | **Matched NHW, n=152** | | **Matched Asian, n=81** | | **p-value^a^** | |
| --- | --- | --- | --- | --- | --- | --- | --- | --- | --- |
| **Donor demographics** | | | | | | | | | |
| Donor age (y) | | | Mean ± SD | 31.7±11.0 | | 32.9±11.2 | | 0.421 | |
| Donor gender | | | Female (%) | 35 (23.0%) | | 23 (28.4%) | | 0.367 | |
|  |  |  | Male (%) | 117 (77.0%) | | 58 (71.6%) | |  |  |
| Body surface area | | | Mean ± SD | 1.94±0.20 | | 1.93±0.21 | | 0.847 | |
| Donor race | | | White (%) | 129 (84.9%) | | 67 (82.7%) | | 0.041 | |
|  |  |  | Black (%) | 21 (13.8%) | | 8 (9.9%) | |  |  |
|  |  |  | Others (%) | 2 (1.3%) | | 6 (7.4%) | |  |  |
| Left ventricular ejection fraction (%) | | Mean ± SD | | | 62.8±7.6 | | 62.5±6.2 | |  |
| Creatinine (mg/dL) | | Mean ± SD | | | 1.04±0.59 | | 0.99±0.41 | |  |
|  | | Anoxia | | | 49 (32.2%) | | 23 (28.4%) | |  |
| **Cause of death** | | Cerebrovascular accident | | | 17 (11.2%) | | 20 (24.7%) | | 0.029 |
|  | | Head trauma | | | 85 (55.9%) | | 36 (44.4%) | |  |
|  | | Other | | | 1 (0.7%) | | 2 (2.5%) | |  |
| **Donor comorbidity history** | | | | | | | | | |
| Cancer | | | Yes (%) | 4 (2.6%) | | 2 (2.5%) | | 0.763 | |
| Diabetes | | | Yes (%) | 2 (1.3%) | | 2 (2.5%) | | 0.624 | |
| Hypertension | | | Yes (%) | 13 (8.6%) | | 13 (16.1%) | | 0.196 | |
| Myocardial infarction | | | Yes (%) | 2 (1.3%) | | 0 (0.0%) | | 0.445 | |

^a^p-values from the Chi-squared test, Fisher's exact test, two-sample t-test, or Wilcoxon rank sum test

NHW, non-Hispanic White; SD, standard deviation

**Supplementary Figure 1.** Kaplan–Meier survival analysis after heart-kidney transplantation in the prematched cohort stratified by the eGFR score

Kaplan–Meier survival estimates show no statistically significant difference in the long-term survival rates between the NHW and Asian groups across all eGFR subgroups and time points (A. eGFR > 45 mL/min/1.73m², p=0.464; B. eGFR 30–45 mL/min/1.73m², p=0.666; C. eGFR < 30 mL/min/1.73m², p=0.471). Log-rank test was used to compare survival between the groups. Censoring marks are indicated.

NHW, non-Hispanic White

**Supplementary Figure 2.** SMDs of NHW vs. Asian recipients

NHW, non-Hispanic White; SMD, standardized mean difference
