# Supplementary figures and images for: Simultaneous heart-kidney transplantation outcomes in Asian populations in the United States: A united network for organ sharing database study
Source: JHLT Open. 2025 Aug 5;10:100364. doi: 10.1016/j.jhlto.2025.100364 (PMC12396451; doi:10.1016/j.jhlto.2025.100364)

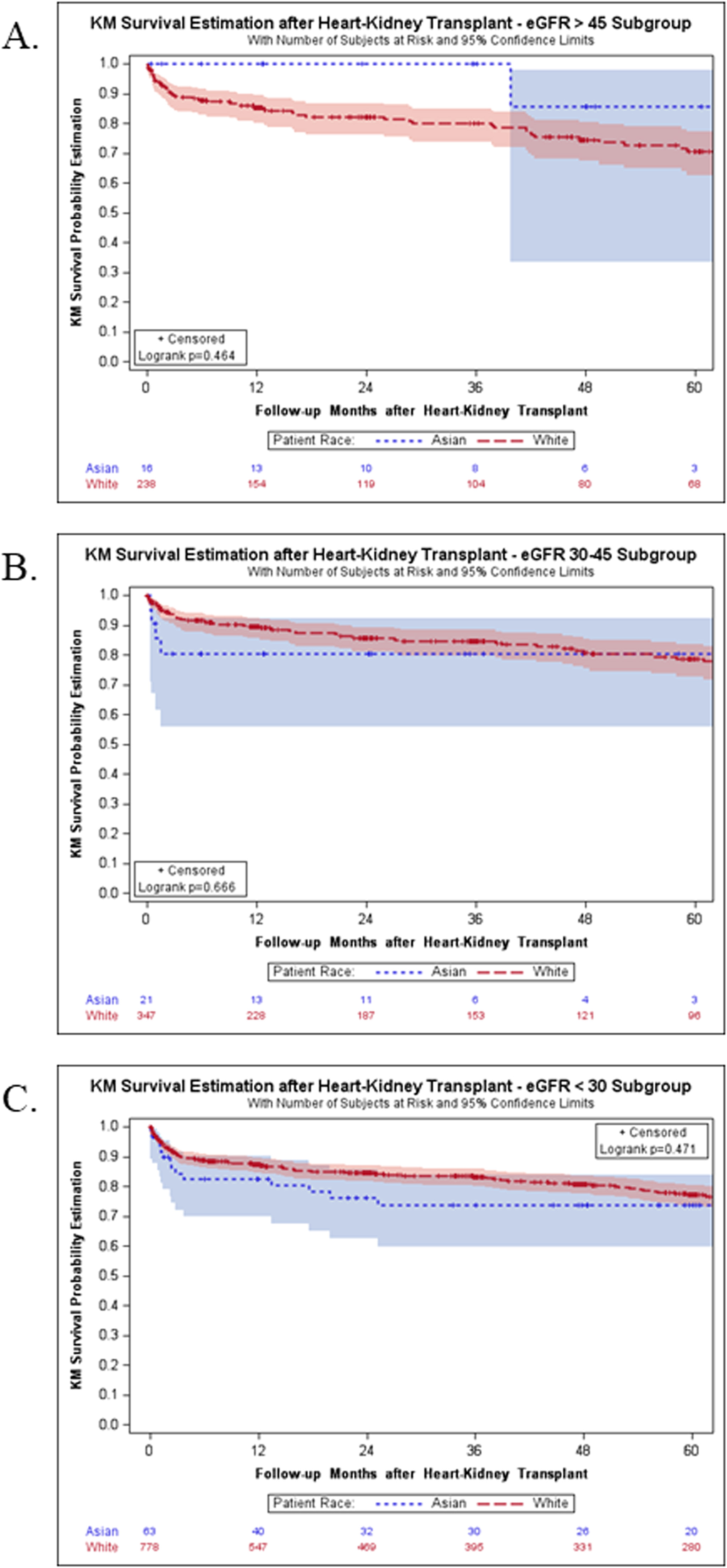

Supplement: Supplementary file 2 — Supplemental material [file mmc2.jpg]

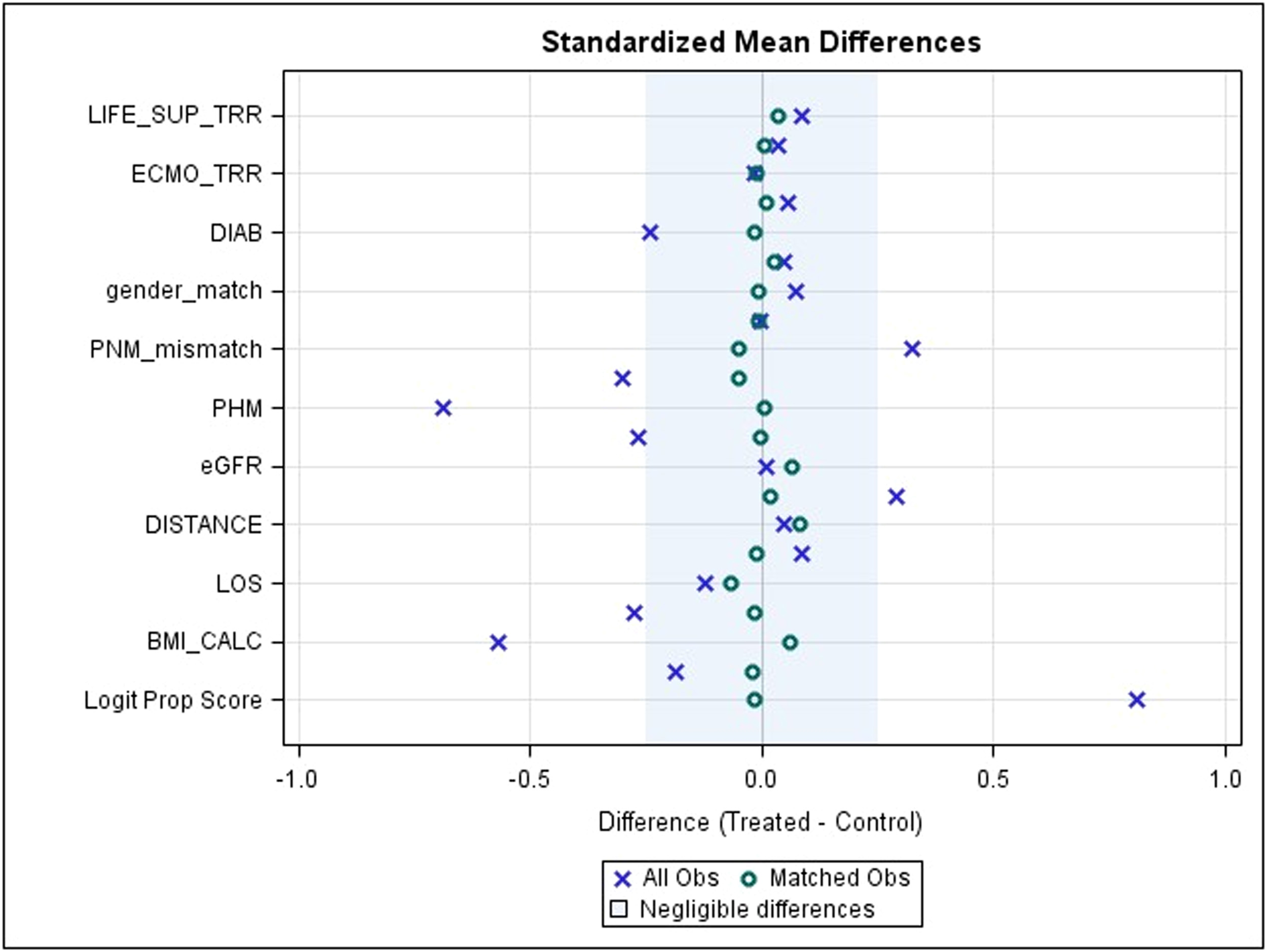

Supplement: Supplementary file 3 — Supplemental material [file mmc3.jpg]
